# Efficacy analysis of ultrashort wave therapy as adjunctive treatment for pediatric patients with macrolide-resistant Mycoplasma pneumoniae pneumonia

**DOI:** 10.3389/fcimb.2025.1712824

**Published:** 2025-11-24

**Authors:** Shuqi Wang, Yanjun Wang

**Affiliations:** Department of Pediatrics, Affiliated Hospital of Chifeng University, Chifeng, China

**Keywords:** Mycoplasma pneumonia, children, macrolide resistance, ultrashort wave therapy, adjunctive treatment, pulmonary lesions

## Abstract

**Background:**

The incidence of macrolide-resistant Mycoplasma pneumoniae pneumonia (MRMPP) has been increasing in children, resulting in prolonged illness and complications. This study aimed to evaluate the effect of ultrashort-wave therapy (USWT) as a second-line therapy in patients with MRMPP.

**Methods:**

This is a retrospective controlled practice clinical study of 112 children hospitalized with MRMPP, assigned to a control group (n = 75) who received standard therapy, or a USWT group (n = 37), who received standard therapy plus USWT. The groups were assigned based on parental preference, not randomization. The primary outcomes studied were length of stay, pulmonary lesion resolution, and new lesion rate; a secondary analysis investigated time to introduce USWT.

**Results:**

The baseline clinical characteristics were similar between the two groups, with the exception of the mean length of stay. The length of stay was significantly longer for the USWT group (10.9 ± 3.1 vs. 9.4 ± 2.4 days, p = 0.01). The resolution of lesions was significantly higher (97.3 vs. 72.0%, p < 0.01), and new lesion rates were lower in the USWT group (16.2 vs. 29.3%), but not significant (p = 0.20). The multivariate analysis confirmed that USWT and severity of pneumonia were significant independent variables affecting lung length of stay (p <0.01; p <0.001).

**Conclusions:**

USWT as adjunctive therapy for children with MRMPP significantly improved resolution of pulmonary lesions, while also being associated with longer length of stay. These findings suggest that USWT may be a supportive, non-pharmacologic adjunctive therapy to improve recovery from antibiotic-resistant pediatric pneumonia.

## 1 Introduction

*Mycoplasma pneumoniae* pneumonia (MPP) is one of the most common causes of community-acquired pneumonia in children, accounting for 10–40% of all pediatric pneumonia cases worldwide (Hu et al., 2023). While traditionally considered a mild and self-limiting infection, MPP has demonstrated changing epidemiological and clinical patterns in recent years, with increasing reports of severe and refractory cases (Biagi et al., 2021). Of particular concern is the global rise in macrolide-resistant *Mycoplasma pneumoniae* pneumonia (MRMPP) strains, which has significantly complicated treatment approaches and clinical outcomes (Principi and Esposito, 2013).

Macrolides, particularly azithromycin, have long been the first-line antibiotics for MPP treatment due to their excellent tissue penetration, anti-inflammatory properties, and favorable safety profile in pediatric populations (Wang et al., 2022). However, macrolide resistance rates have increased dramatically over the past decade, reaching alarming levels of 80–90% in some Asian countries, including China, Japan, and South Korea (Zhao et al., 2013; Dai et al., 2021). This resistance is primarily attributed to point mutations in domain V of the 23S rRNA gene, which prevent macrolide binding and render these antibiotics ineffective (Kawakami et al., 2021).

The clinical management of MRMPP poses significant challenges for pediatricians. Children with MRMPP typically experience prolonged fever, persistent cough, and more severe radiological findings compared to those with macrolide-sensitive infections (Zhou et al., 2014). The disease course is often protracted, leading to extended hospitalization, increased risk of extrapulmonary complications, and substantial healthcare costs (Nakamura et al., 2021). While alternative antibiotics such as fluoroquinolones and tetracyclines demonstrate activity against resistant strains, their use in children is limited by safety concerns, including potential adverse effects on cartilage development and tooth discoloration (Bradley et al., 2011).

Given these therapeutic limitations, there is growing interest in adjunctive treatments that can ameliorate the inflammatory response and accelerate recovery in children with MRMPP. The pathogenesis of MPP involves not only direct cytotoxicity but also significant host immune responses, with excessive inflammation contributing substantially to lung injury and clinical manifestations (Zhang et al., 2022). This understanding has prompted exploration of various immunomodulatory approaches as adjunctive therapies.

Corticosteroids have shown some benefit in managing severe MPP cases by suppressing excessive inflammation (Tong et al., 2022). However, concerns about immunosuppression, metabolic effects, and potential impacts on growth have limited their routine use, particularly for prolonged courses (Lee et al., 2006). This has created a need for alternative adjunctive therapies with favorable safety profiles that can effectively modulate the inflammatory response without compromising host defense mechanisms.

Ultrashort-wave therapy (USWT) represents a non-pharmacological approach that has been used in various inflammatory conditions for its anti-inflammatory and tissue-healing properties (Giombini et al., 2007). The therapy utilizes high-frequency electromagnetic waves (typically 27.12 MHz) that generate thermal effects in tissues, potentially improving local circulation, enhancing phagocytic activity, and modulating inflammatory mediators (Li et al., 2023). The non-invasive nature and minimal adverse effects of USWT make it an attractive option for pediatric applications.

In respiratory conditions, USWT has been hypothesized to reduce local inflammation, improve pulmonary circulation, and enhance the penetration of antibiotics into affected lung tissues (Wang et al., 2021). Previous studies have suggested potential benefits of USWT in adult patients with various respiratory infections, including pneumonia and bronchitis (Li et al., 2024). However, high-quality clinical evidence regarding its efficacy in pediatric respiratory infections, particularly MRMPP, remains limited.

The physiological mechanisms underlying potential USWT benefits in MRMPP may involve several pathways. The thermal effects generated by electromagnetic waves can increase local tissue temperature by 2–3 °C, enhancing blood flow and lymphatic drainage in inflamed pulmonary tissues (Yang et al., 2024). This improved circulation could facilitate the removal of inflammatory mediators and cellular debris, accelerating the resolution of inflammation. Additionally, the increased tissue temperature may enhance phagocytic activity of macrophages and neutrophils, potentially improving clearance of *Mycoplasma* organisms (Goats, 1990).

Furthermore, USWT has been shown to modulate the production of cytokines involved in the inflammatory cascade, including tumor necrosis factor-alpha (TNF-α), interleukin-6 (IL-6), and interleukin-1 beta (IL-1β), which are known to be elevated in MPP infections (Yang et al., 2019). By attenuating excessive cytokine responses, USWT might help mitigate the immunopathological damage characteristic of severe MPP.

Another proposed mechanism involves the enhancement of pulmonary surfactant production and function, which can be compromised during pneumonia (Chaudhuri et al., 2024). Improved surfactant activity could potentially reduce atelectasis and improve ventilation–perfusion matching in affected lung segments. Moreover, the increased local temperature and blood flow might enhance antibiotic penetration in infected lung tissues, potentially improving efficacy even in the context of macrolide resistance (Quarato et al., 2023).

Despite these physiological rationales, there is a paucity of controlled clinical studies evaluating the efficacy of USWT specifically in pediatric MRMPP. Most existing literature consists of small case series or observational studies with methodological limitations that preclude definitive conclusions. Additionally, questions remain regarding optimal treatment protocols, including timing of initiation, duration, frequency, and intensity of USWT sessions for maximum benefit.

Given the increasing prevalence of MRMPP and the limited therapeutic options available for pediatric patients, there is an urgent need for evidence-based adjunctive treatments that can improve clinical outcomes. If proven effective, USWT could represent a valuable addition to the therapeutic armamentarium for managing MRMPP in children, potentially reducing disease burden and accelerating recovery.

Therefore, we conducted a retrospective controlled clinical study to evaluate the adjunctive efficacy of USWT in pediatric MRMPP, clearly defining its impact on hospital stay, pulmonary lesion resolution, and timing of initiation.

## 2 Methods

### 2.1 Study design and patient selection

This study was a non-randomized controlled clinical study with a retrospective design. Group assignment was based on parental preferences after full exploration of treatment options. The non-randomized design may produce selection bias, which is noted as a limitation in this study. Because this study was a retrospective exploratory study, *a priori* sample-size estimation was not completed nor was sensitivity analysis employed.

This retrospective study analyzed clinical data from pediatric patients with Mycoplasma pneumonia treated at the Department of Pediatrics between September 2023 and December 2024. Pediatric patients aged 2–8 years with radiologically confirmed pneumonia and clinical suspicion of Mycoplasma pneumoniae infection were initially screened for eligibility. Mycoplasma pneumoniae infection was confirmed by positive polymerase chain reaction (PCR) detection of M. pneumoniae DNA in throat swabs. Macrolide resistance was clinically defined as persistence of fever (≥38.0 °C) for at least 72 hours despite appropriate macrolide therapy (azithromycin 10 mg/kg/day for 3–5 days).

Severity classification was based on the Chinese Pediatric CAP Guidelines (2021): severe cases were defined by any of the following—respiratory rate > 60/min (age < 2 years) or > 50/min (≥ 2 years), SpO_2_ < 92% on room air, multilobar infiltration on imaging, or need for oxygen supplementation.

Exclusion criteria included; (1) underlying chronic pulmonary diseases such as asthma, bronchopulmonary dysplasia, or congenital lung malformations; (2) immunodeficiency disorders; (3) history of adverse reactions to electromagnetic therapy; (4) presence of electronic medical devices (e.g., pacemakers); (5) severe extrapulmonary complications requiring intensive care; and (6) inability to comply with the study protocol or parental refusal to participate.

Eligible patients were allocated to either the control group (standard treatment) or the ultrashort wave therapy group (standard treatment plus ultrashort wave therapy) based on parental preference after detailed explanation of the treatment options. While not a randomized allocation, this approach was adopted to address ethical considerations and parental concerns regarding a novel treatment modality in pediatric patients.

### 2.2 Treatment protocols

#### 2.2.1 Standard treatment

All patients in both groups received the following standard treatment regimen:

Antibiotic therapy: Given the clinical suspicion of macrolide resistance, patients were treated with appropriate alternative antibiotics based on age and clinical severity. Antibiotic selection followed national pediatric pneumonia guidelines and hospital antimicrobial-stewardship recommendations, considering age, hepatic/renal function, allergy history, and local resistance patterns. Tetracyclines were avoided in children < 8 years, and quinolones were reserved for refractory cases. Antibiotic regimens were adjusted based on clinical response and continued until clinical improvement was achieved (typically resolution of fever for at least 48–72 hours and significant improvement in respiratory symptoms).

Corticosteroid therapy: Methylprednisolone (1–2 mg/kg/day, divided into two doses) was administered intravenously to patients with severe clinical manifestations, including persistent high fever, significant respiratory distress, or extensive radiological involvement. The duration of corticosteroid therapy was determined by clinical response, with a typical course of 5–7 days followed by gradual tapering.

Supportive care: This included adequate hydration, oxygen supplementation if required (to maintain SpO_2_ >92%), antipyretics for fever control (acetaminophen or ibuprofen as needed), and chest physiotherapy to facilitate mucus clearance.

Bronchodilators: Inhaled β2-agonists (salbutamol or tebutaline) were administered to patients with wheezing or evidence of bronchospasm.

#### 2.2.2 Ultrashort wave therapy protocol

In addition, Ultrashort-wave therapy was delivered using a medical-grade ultrashort-wave therapeutic apparatus (Model: Dier-501, Manufacturer: Dier Electronic Technology Co., Ltd., Guangzhou, China). The device was calibrated weekly according to the manufacturer’s protocol to ensure consistent output power and frequency accuracy. Controls did not undergo sham electrode placement. The therapy was delivered according to the following protocol:

Preparation: The patient was positioned in a comfortable supine position with the treatment area (bilateral lung fields) exposed. Metallic objects (jewelry, coins, etc.) were removed from the treatment area.

Device settings: The ultrashort wave therapy device was set to operate at a frequency of 27.12 MHz with an output power of 200 watts, using the continuous wave mode.

Application: Two electrode plates (15 cm in diameter) were positioned anteriorly and posteriorly over the lung fields, the placement of the electrode uses the opposing method, with a distance of 1–2 cm between the electrode and the skin, and a cotton pad is placed between. The treatment duration was 15 minutes per session, conducted once or twice daily (morning and afternoon, with at least 6 hours between sessions).

Treatment course: Ultrashort wave therapy was initiated based on clinical judgment, typically after the patient had been on standard treatment for 2–5 days without adequate response. The therapy was continued until significant clinical improvement was observed (resolution of fever, improved respiratory symptoms, and radiological improvement), typically for 5–7 days.

Safety monitoring: Patients were continuously monitored during the therapy sessions for any adverse effects, including skin reactions, discomfort, or changes in vital signs. The therapy was to be immediately discontinued if any adverse events occurred.

### 2.3 Outcome measures

#### 2.3.1 Primary outcomes

Hospital length of stay: Number of days from admission to discharge.

Pulmonary lesion reduction rate: Radiological improvement was scored by two blinded radiologists using a semi-quantitative 4-point scale (0 = no change; 1 = <25% reduction; 2 = 25–49%; 3 = ≥50%). Discrepancies were resolved by consensus.

New lesion occurrence rate: Defined as new pulmonary infiltrates in previously unaffected lung segments during hospitalization.

#### 2.3.2 Secondary outcomes and exploratory analyses

Secondary outcomes included:

Duration of fever after admission: Defined as the number of days with body temperature ≥38.0 °C after hospitalization.

Duration of corticosteroid therapy: Measured in days from initiation to discontinuation.

Timing of ultrashort wave therapy initiation: For the ultrashort wave therapy group, the relationship between the timing of therapy initiation (defined as the number of days after admission) and clinical outcomes was explored. Early initiation was defined as starting therapy within the first 5 days of admission, while late initiation was defined as starting after day 5.

Adverse events: Any adverse events potentially related to ultrashort wave therapy were recorded, including skin reactions, discomfort, burns, or electromagnetic interference with other medical devices.

### 2.4 Data collection and monitoring

Demographic data, clinical parameters, laboratory findings, radiological results, and treatment details were collected using standardized case report forms. Key variables included:

Demographic information: Age, gender, weight, height, and pre-existing conditions.

Clinical parameters: Vital signs (temperature, heart rate, respiratory rate, blood pressure, oxygen saturation), respiratory symptoms (cough, sputum, dyspnea, chest pain), and physical examination findings.

Laboratory tests: Complete blood count, C-reactive protein (CRP), procalcitonin, liver and kidney function tests, and PCR results.

Radiological findings: Features, extent, and location of pulmonary infiltrates on chest radiographs or CT scans.

Treatment details: Antibiotics (type, dose, duration), corticosteroids (dose, duration), and ultrashort wave therapy parameters (timing, frequency, duration).

Patients were monitored daily during hospitalization, with clinical assessments performed by pediatricians unaware of the study objectives. Radiological evaluations were conducted at admission and before discharge, with additional examinations performed as clinically indicated.

### 2.5 Statistical analysis

All statistical analyses were performed using R software (version 4.1.0; R Foundation for Statistical Computing, Vienna, Austria) with packages *stats* and *car*. Regression coefficients (β) are presented with standard errors and 95% confidence intervals (CIs). Multiple comparisons were not adjusted due to the exploratory nature of the study; this limitation is discussed in the Discussion section.

Continuous variables were expressed as means ± standard deviations or medians with interquartile ranges (IQR), depending on the distribution. Categorical variables were presented as counts and percentages.

For between-group comparisons, Student’s t-test or Welch’s t-test was used for normally distributed continuous variables, while the Mann-Whitney U test was used for non-normally distributed continuous variables. The Shapiro-Wilk test was used to assess the normality of continuous variables. Chi-square test or Fisher’s exact test was used for categorical variables as appropriate.

To adjust for potential confounding factors, multivariate linear regression analysis was performed for hospital length of stay, incorporating treatment group, gender, age, disease severity, duration of fever before admission, previous azithromycin use, and duration of azithromycin treatment before admission as covariates.

The relationship between the timing of ultrashort wave therapy initiation and hospital stay was assessed using Pearson’s correlation analysis and by comparing early versus late initiation groups using Welch’s t-test.

A p-value <0.05 was considered statistically significant for all analyses.

## 3 Results

### 3.1 Baseline characteristics of patients

A total of 112 pediatric patients with clinically defined macrolide-resistant Mycoplasma pneumonia were included in the analysis, with 75 patients in the control group and 37 patients in the ultrashort wave therapy group. The baseline demographic and clinical characteristics of both groups are summarized in **Table 1**.

**Table 1:** Comparison of baseline characteristics between the control group and the ultrashort wave therapy group.

Characteristic	Control group (n=75)	Ultrashort wave group (n=37)	p-value
Gender, n (%)	Female 37 (49.3) Male 38 (50.7)	Female 20 (54.1) Male 17 (45.9)	0.788
Age, months (median [IQR])	72.00 [57.00, 84.00]	72.00 [60.00, 84.00]	0.906
Disease severity, n (%)	Non-severe 66 (88.0)	Non-severe	1.000
Hospital days (median [IQR])	9.00 [7.50, 11.00]	10.00 [9.00, 12.00]	0.007*
Fever days before admission (median [IQR])		4.00 [3.00, 6.00]	0
Azithromycin before admission, n (%)	No 6 (8.0) Yes 69 (92.0)	No 3 (8.1) Yes 34 (91.9	1.000
Azithromycin days before admission (median [IQR])	3.00 [3.00, 4.00]	4.00 [3.00, 5.00]	0.074
Hormone days (median [IQR])	5.00 [5.00, 6.00]	5.00 [5.00, 7.00]	0.288

*Statistically significant (p<0.05) IQR: Interquartile range.

Hospital days were analyzed separately as an outcome variable and, therefore, excluded from baseline comparisons.

**Figure 1:**
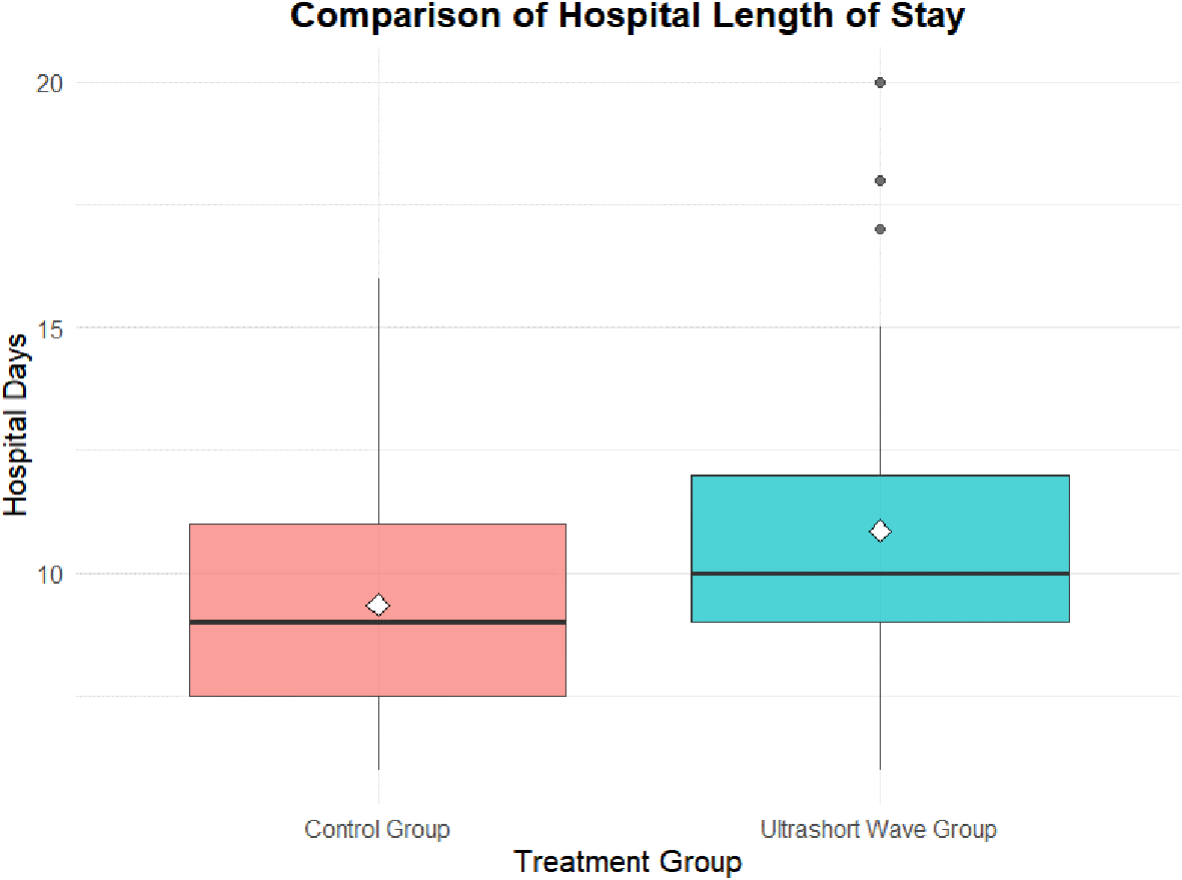
Comparison of hospital length of stay between groups. Box-and-whisker plot illustrating hospital length of stay in the control and ultrashort-wave therapy (USWT) groups. The median hospitalization was significantly longer in the USWT group (10.0 days, IQR 9.0–12.0) compared with the control group (9.0 days, IQR 7.5–11.0; *p = 0.007*).

**Figure 2:**
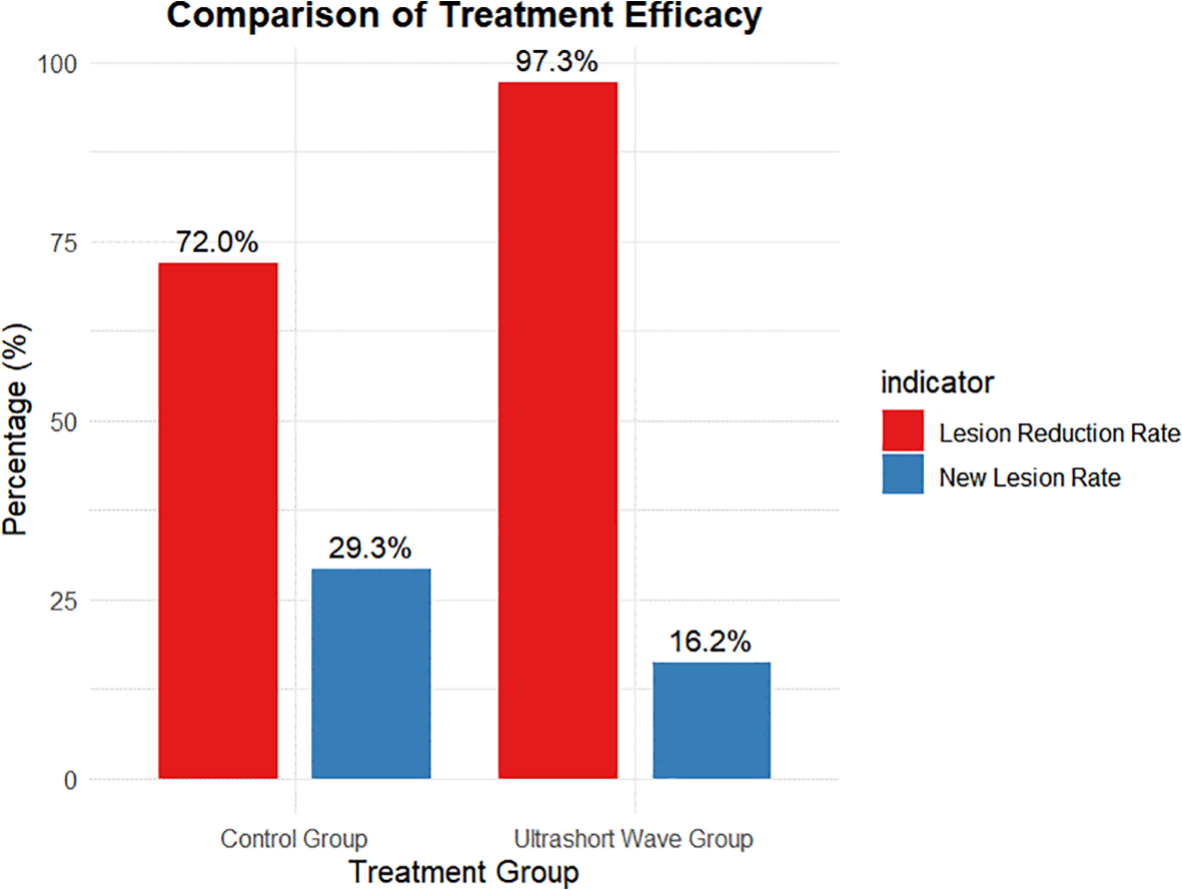
Comparison of pulmonary lesion reduction between groups. Bar chart showing the proportion of patients achieving ≥50% radiologic lesion reduction before discharge. The USWT group exhibited a significantly higher lesion-reduction rate (97.3%) compared to the control group (72.0%; *p = 0.004*), indicating enhanced radiologic recovery associated with adjunctive USWT.

**Figures 1**, **2** (gender and age distribution) were removed due to redundancy with **Table 1**.

### 3.2 Hospital length of stay analysis

The hospital length of stay was significantly longer in the USWT group compared to the control group. The median stay was 9.0 days (IQR 7.5–11.0) for the control group versus 10.0 days (IQR 9.0–12.0) for the USWT group (p = 0.007). The mean stay was 9.36 ± 2.35 days vs. 10.86 ± 3.11 days, respectively (p = 0.012) shown in **Figure 1**.

Multivariate linear regression identified USWT (β = 1.394, p = 0.008) and disease severity (β = 3.617, p < 0.001) as independent predictors of longer hospitalization after adjustment for confounding factors **Table 2**.

**Table 2:** Multivariate linear regression analysis of factors associated with hospital length of stay.

Variable	Estimate	Standard error	t value 95% CI	p-value
(Intercept)	5.779	1.608	3.595 2.59 – 8.97	0.000***
Treatment group (Ultrashort Wave vs. Control)	1.394	0.515	2.706 0.37 – 2.42	0.008**
Gender (Male vs. Female)	-0.081	0.489	-0.165 −1.05 – 0.89	0.869
Age (months)	0.011	0.015	0.715 −0.02 – 0.04	0.476
Disease severity (Severe vs. Non-severe)	3.617	0.897	4.034 1.84 – 5.39	0.000***
Fever days before admission	0.043	0.122	0.351 −0.20 – 0.29	0.727
Azithromycin before admission (Yes vs. No)	1.940	1.278	1.518 −0.60 – 4.48	0.132
Azithromycin days before admission	0.135	0.258	0.522 −0.38 – 0.65	0.603

Significance codes: ***p<0.001, **p<0.01. Residual standard error: 2.495 on 104 degrees of freedom. Multiple R-squared: 0.205, Adjusted R-squared: 0.151. F-statistic: 3.819 on 7 and 104 DF, p-value: 0.001.

The model accounted for approximately 20% of the variance (R² = 0.205), indicating that additional unmeasured clinical factors likely contributed to hospital-stay duration.

The multivariate analysis confirmed that treatment with ultrashort wave therapy was independently associated with longer hospital stays (coefficient: 1.394, p=0.008) after adjusting for other variables. Disease severity was also identified as a significant predictor of hospital stay length, with severe cases having approximately 3.617 days longer hospitalization (coefficient: 3.617, p=0.000). The model explained approximately 20.5% of the variance in hospital length of stay (R-squared: 0.205), and the overall regression was statistically significant (F-statistic: 3.819, p=0.001). Other factors, including gender, age, duration of fever before admission, previous azithromycin use, and duration of azithromycin treatment before admission, did not significantly influence hospital length of stay in this model.

The model explained approximately 20.45% of the variance in hospital length of stay (R-squared: 0.2045), and the overall regression was statistically significant (F-statistic: 3.819, p=0.001).

### 3.3 Treatment efficacy analysis

#### 3.3.1 Pulmonary lesion reduction rate

The pulmonary lesion reduction rate, defined as the proportion of patients showing significant radiological improvement before discharge, was significantly higher in the ultrashort wave therapy group. As shown in **Figure 2**, 97.3% (36/37) of patients in the ultrashort wave therapy group demonstrated a significant reduction in pulmonary lesions compared to 72.0% (54/75) in the control group. This difference was statistically significant based on chi-square analysis with Yates’ continuity correction (χ² = 8.507, df = 1, p=0.004).

The contingency table for lesion reduction is presented in **Table 3**.

**Table 3:** Contingency table for pulmonary lesion reduction by treatment group.

Treatment group	No reduction	Reduction	Total
Control Group	21 (28.0%)	54 (72.0%)	75
Ultrashort Wave Group	1 (2.7%)	36 (97.3%)	37
Total	22	90	112

χ² = 8.5066, df = 1, p=0.004.

#### 3.3.2 New lesion occurrence rate

The occurrence of new pulmonary lesions during hospitalization was lower in the ultrashort wave therapy group, though the difference did not reach statistical significance. As shown in **Figure 1**, new lesions developed in 16.2% (6/37) of patients in the ultrashort wave therapy group compared to 29.3% (22/75) in the control group (χ² = 1.628, df = 1, p=0.202). The contingency table for new lesion occurrence is presented in **Table 4**.

**Table 4:** Contingency table for new lesion occurrence by treatment group.

Treatment group	No new lesions	New lesions	Total
Control Group	53 (70.7%)	22 (29.3%)	75
Ultrashort Wave Group	31 (83.8%)	6 (16.2%)	37
Total	84	28	112

χ² = 1.628, df = 1, p=0.202.

#### 3.3.3 Summary of treatment outcomes

A summary of the main treatment outcomes comparing the control group and ultrashort wave therapy group is presented in **Table 5**.

**Table 5:** Summary of treatment outcomes by group.

Indicator	Control group (n=75)	Ultrashort wave group (n=37)	p-value
Sample size	75	37	–
Mean age (months)	69.2	69.8	0.906
Hospital days (mean ± SD)	9.4 ± 2.4	10.9 ± 3.1	0.012*
Lesion reduction rate	72.0%	97.3%	0.004*
New lesion occurrence rate	29.3%	16.2%	0.202

*Statistically significant (p<0.05) SD: Standard deviation.

### 3.4 Timing of ultrashort wave therapy analysis

For patients in the ultrashort wave therapy group, we examined whether the timing of therapy initiation influenced clinical outcomes, particularly hospital length of stay. The median day of ultrashort wave therapy initiation was 5.00 days after admission (IQR: 3.00-6.00), with a range from 2 to 13 days.

**Figure 3:**
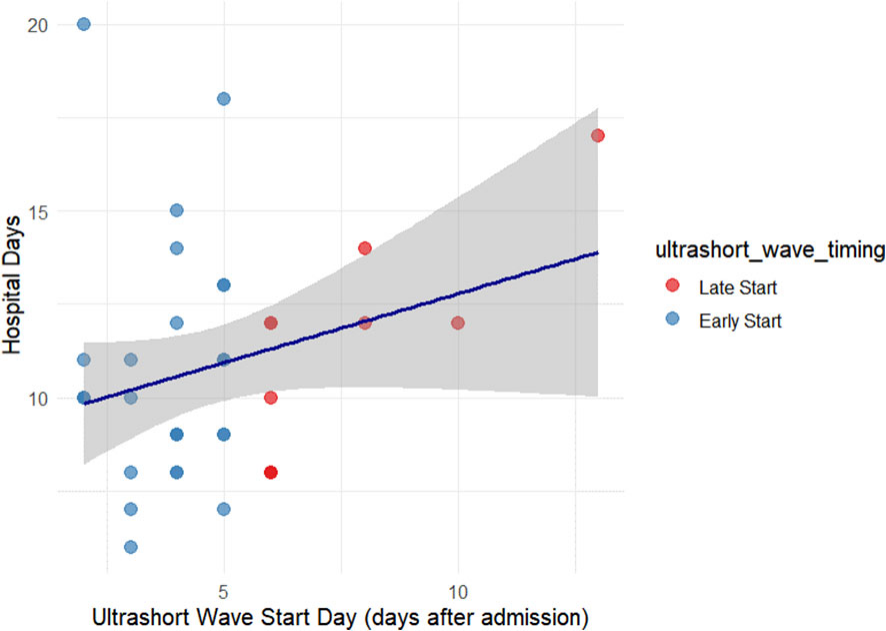
Relationship between ultrashort-wave therapy initiation timing and hospital stay. Scatter plot depicting the correlation between the initiation day of USWT and total hospital stay among treated patients. No statistically significant correlation was observed (*r = 0.268, p = 0.109*). The dashed trend line shows a mild, non-significant upward slope, suggesting that treatment timing had minimal effect on hospitalization duration.

Pearson’s correlation between initiation day and hospital stay showed no statistically significant correlation (r = 0.268, p = 0.109). This relationship is illustrated in **Figure 3**.

To further explore the impact of treatment timing, patients in the ultrashort wave therapy group were categorized into early initiation (≤5 days after admission, n=27) and late initiation (>5 days after admission, n=10) subgroups. The mean hospital stay was 10.70 ± 2.87 days in the early initiation group and 11.30 ± 3.80 days in the late initiation group. This difference was not statistically significant (t = 0.538, df = 17.765, p = 0.597).

The detailed results of the comparison between early and late ultrashort wave therapy initiation are presented in **Table 6**.

**Table 6:** Comparison of hospital stay between early and late ultrashort wave therapy initiation.

Timing of therapy initiation	n	Hospital days (mean ± SD)	95% CI	p-value
Early Initiation (≤5 days)	27	10.70 ± 2.87	9.56-11.85	0.597
Late Initiation (>5 days)	10	11.30 ± 3.80	8.56-14.04	

CI, Confidence interval; SD, Standard deviation.

## 4 Discussion

This research investigated ultrashort-wave therapy (USWT) as an adjunctive therapy for children with macrolide-resistant Mycoplasma pneumoniae pneumonia (MRMPP). It was found that USWT was associated with improvement in the resolution of the extent of radiologic lesions in relation to the standard therapy group, although the length of stay in the hospital was longer. These results provide information about the potential and limitations of the use of this modality of physical therapy in children with antibiotic-resistant pneumonia.

The considerable improvement in pulmonary lesion resolution seen with USWT is consistent with previous studies that show that electrophysical or high frequency physiotherapies can increase *local* circulation, reduce inflammation, and assist with tissue repair (Laufer and Dar, 2012; Hosny et al., 2024). The heat generated from the transmission of ultrashort waves will enhance vascular permeability and enhance microcirculatory perfusion in the whole lung, which can facilitate clearance of inflammatory exudates and restoration of lung architecture (Tsai and Hamblin, 2017). The mechanisms of USWT may, at least in part, explain the significantly greater rate of radiologic improvement in the adjunctive USWT therapy group.

At a molecular level, USWT has been shown to downregulate pro-inflammatory mediators like tumor necrosis factor-alpha (TNF-α), interleukin-1β (IL-1β) and interleukin-6 (IL-6), which can reduce cytokine-induced alveolar injury (Narita et al., 2000; Kumaran and Watson, 2015). This modulation of inflammation likely results in faster resolution of lung lesions and less severe radiography in our population with MRMPP, since injury is often immune-mediated. Similar physiological benefits of USWT have been described in a variety of other pediatric pulmonary conditions, suggesting it may represent a safe, non-pharmacological adjunctive therapy in children (Yang et al., 2021).

While this study did not demonstrate a statistically significant difference in new lesion formation while receiving USWT compared to standard therapy, it may suggest that, despite the limited efficacy of USWT, there may be a trend resulting in a secondary protective effect against disease progression. MRMPP often shows a pattern of stepwise progression with different lung lobes involved sequentially, and while theoretical, the physical modalities involved in stabilizing inflammatory responses may diminish that sequential progression (Chen et al., 2024).

However, the longer hospitalization observed among patients who’ve received USWT (ultrasound wave therapy) should be interpreted with caution, as, in this retrospective, non-randomized study, and even though the analysis provided the grouping they received in the study to be potentially biased thereby interpreting the USWT and longer hospitalization (rather than necessarily being causal associations of USWT and longer hospitalization) is important to note. The method that they were allocated to treatment, taking into account parental-preference for treatment administration, may be biased towards children who had more severe or more refractory disease to typical treatments, which would inherently cause them to potentially have longer hospitalization. Also, in this cohort, not only did improvement need to be considered in both the clinical status on physical assessment but also a radiological basis too - a kid could present with mild symptoms of cough or fatigue, although they would meet criteria on imaging for recovery which may have been delaying discharge (He et al., 2017). Furthermore, children who required in-hospital respiratory therapies (especially equipment-based) likely had longer hospitalizations for logistical rather than medical reasons.

Explaining the limited proportion of the variance in hospital length by the regression model we developed (R²≈0.20), it is very possible hospital length was planned or modulated by other clinical variables, that is clinical variables not measured (such as nutrition, the host immune status, or duration of illness) likely contributed to hospital length - highlighting the multifactorial influence from parameters that would influence management of MRMPP and time to resolution.

We expect the absence of adverse effects of treatment noted in our study is similar to what is being observed in prior safety assessments related to electromagnetic-based physiotherapies in children (Masiá et al., 2005), and the aggregate safety outcome reinforces feasibility regarding the feasibility of using USWT in the pediatric population. It is noted, however, as with any treatment or supportive measure should be administered systematically, and the involvement of continued monitoring in the clinical role would be paramount.

### 4.1 Limitations

We also should note that the parental preference grouping introduces some selection bias for the cohort, along with the possible limitations from the grouping size of the children in the study, as well as the lack of microbiologic results to support or confirm macrolide resistance type. Further, while radiological analysis was assessed by two blinded radiologists, the assessment will still maintain a measure of subjectivity when reporting an outcome. There is also no multiplicity correction in the comparisons that were made, that too could increase the potential for a type I error. Furthermore, the outcome as a retrospective design, and therapy was provided in the hospital, impacted our ability to study the benefit from the outpatient outcome (timing), or any long-term impact of the treatment component.

### 4.2 Future directions

For future studies, the evaluation of treating symptoms duration and oxygen requirement, and likely recurrence rate would be clinical and meaningful outcomes, and beyond just radiological outcome assessment. Additionally, the possible inclusion of objective clinical outcomes methods (such as inflammatory markers or lung function metrics), or cost-effectiveness would also provide a better picture of the value of USWT in treating resistant pediatric pneumonia.

## Data Availability

The original contributions presented in the study are included in the article/supplementary material. Further inquiries can be directed to the corresponding author.
